# Reflections on The Global Influenza Surveillance and Response System (GISRS) at 65 Years: An Expanding Framework for Influenza Detection, Prevention and Control

**DOI:** 10.1111/irv.12511

**Published:** 2018-02-19

**Authors:** Arnold S. Monto

**Affiliations:** ^1^ Department of Epidemiology School of Public Health University of Michigan Ann Arbor MI USA

The history of influenza as a global health concern goes back centuries if not millennia. That history is mainly related to pandemics recognized long before the causative viruses had been identified.^1^ Their spread respected no borders, making them a global concern.^2^ The history of seasonal influenza is much shorter and less clear‐cut, again starting well before the viruses were identified.^3^ The disease was recognized in large part because of the characteristic illnesses occurring over a relatively short period in the colder parts of the world. While the widespread nature of influenza outbreaks was known, the recognition was largely limited to a number of countries, mainly in temperate regions.^4^ Burden of disease was well recognized with estimates based on the methods that did not need to rely on virus identification of individual cases, but rather on the occurrence of the illnesses of certain characteristics in periods with known virus circulation.^5^, ^6^ That recognition led to the development of effective vaccines, starting in the 1940s.^7^ Very quickly it became clear that changes in the influenza virus would make the vaccine ineffective unless it was updated regularly to reflect viruses in circulation and that circulation was global, not limited to a single country or region.

In much of the rest of the world at the time, the presence of the virus as a major cause of year‐to‐year illness was typically not recognized. This was in contrast to pandemics, which because of the large number of cases of disease occurring over a limited time period were impossible to ignore. Only because of the programs seeking to identify the activity and characteristics of influenza viruses globally was there a beginning realization that the viruses were not only present in tropical countries but actually spread for much longer periods of time.^8^ However, because of the lack of sharp seasonality, burden could not be estimated in the same way as in the temperate zones. The development of the reverse‐transcriptase polymerase chain reaction (PCR) technique made identification of actual infection easier, making it possible to define the periods of spread accurately, a necessity for determining impact when there was not sharp seasonality.^9^

Studies have begun to confirm the major impact of non‐pandemic influenza not only in the countries where it was already partially recognized but also in much of the rest of the world, which until recently was all blank areas. Determination of burden has become one of the many activities at the World Health Organization (WHO) dedicated to influenza and its control. It is appropriate that this issue has come out in 2017, a year which marks the 65th anniversary of the antecedents of the Global Influenza Surveillance and Response System (GISRS). Without the system, whose predecessors started even before WHO was formally established, none of these activities would be possible, nor would there be an ability to respond to pandemics and to have vaccines formulated for use on an annual basis.

## ESTABLISHMENT OF GLOBALLY COORDINATED INFLUENZA SURVEILLANCE

In 1947, the WHO Interim Committee of the United Nations agreed to begin a Global Influenza Programme (GIP) for the study and control of influenza. An immediate concern was a major outbreak of influenza in Europe and, recognizing influenza virus evolution, the need to identify appropriate viruses for a vaccine against the types of influenza which might be circulating. One year later, the Interim Committee recommended the establishment of the first World Influenza Centre at the National Institute for Medical Research in London along with Regional Centres and Observers. The 38 regional centers, later named National Influenza Centres (NICs), were called upon to participate in the effort. Extensive activity was undertaken to develop plans and coordinate information and virus sharing.

Five years after the establishment of GIP, WHO's Executive Board decided that an influenza surveillance system was needed to inform the methods for disease prevention and control. The Global Influenza Surveillance Network (GISN) was born. The initial focus of GISN, later to be renamed the GISRS, was on standard diagnostic procedures, preparation, and distribution of diagnostic reagents, and the selection and evaluation of appropriate strains for vaccines. Research and training were also part of the overall effort, mainly focused on virus and strain diversity identification. At this point, understanding disease burden was largely restricted to describing seasonal outbreaks in temperate countries experiencing sharp peaks of activity and enumerating influenza‐related hospitalizations and deaths.^5^, ^6^ In the rest of the world, the presence of the virus as a major cause of year‐to‐year illness was typically not recognized.

Global Influenza Surveillance and Response System gained momentum between the 1957 and 1968 pandemics. The growing network of NICs and the Influenza Centres, later to be called Collaborating Centres for Reference and Research on Influenza (WHO CCs), focused on understanding disease activity and characteristics of influenza viruses globally. These efforts confirmed the realization that the viruses were not only present in tropical countries but might circulate for much of the year.^9^

## EXPANSION OF ACTIVITIES AND PREPARATION FOR A PANDEMIC

Although the main focus of GISRS activities in the subsequent years continued to be identification of influenza virus variants for making vaccine composition as close to the circulating strains as possible, there was gradual expansion to other aspects of influenza control. As examples, a system was adopted in 1980 unifying designation of the viruses by hemagglutinin and neuraminidase whatever the source.^10^ Because of molecular studies, the human influenza viruses prevalent from 1918 to 1957, which had been previously termed ASw, A1, and A0, were all designated A(H1N1). This established the basic system of nomenclature used to this day. The antivirals, amantadine, and rimantadine were the subject of a consultation in 1983 as was evaluation of vaccine efficacy in the community in 1987. The former consultation was one of the first examples of expansion of activities into clinical concerns, a trend which has continued.

Surprisingly, even at that point, there was little global work at WHO in determining the burden of influenza on a global level. This was left to the individual countries and regions. An example of regional collaboration was the European Scientific Working Group on Influenza (ESWI). Their work was based on the realization that recommendations for vaccine use and support of research activities were dependent on recognition by governments both that influenza was a cause of significant morbidity and mortality and that interventions could mitigate its effect. Most studies demonstrating disease burden in various population groups and potential reduction by vaccination were still being carried out mainly in the countries where seasonality of influenza was sharp.^11^ Some began to include economic components.^12^

At WHO, there were meetings in 1998 dedicated to influenza surveillance, but the approach was changing, leading to more emphasis on regions with little prior knowledge of influenza activity and its impact. By 2002, WHO's Executive Board urged countries without national influenza vaccine policy to assess disease burden and economic impact of annual influenza epidemics. Concern about a possible severe influenza pandemic drove much of the activity for the rest of that decade with recognition of multiple outbreaks of avian A(H5N1) viruses mainly in Asia, Africa, and even Europe which occasionally involved humans.^13^, ^14^ Recommendations for the development and use of vaccines and antivirals were made as were efforts at preparing for rapid response.

As pandemic influenza is perceived as a threat in all countries, even in those which have had little prior interest in seasonal influenza, this allowed further expansion of efforts to detect influenza viruses to the countries which did not do so on a regular basis. Much of this expansion was possible only because of what can only be described as a technologic breakthrough, the development, and the dissemination of polymerase chain reaction (PCR) technique. Influenza viruses could now easily be identified accurately with high sensitivity and specificity. Global Influenza Surveillance and Response System supported and accelerated the process by the provision of reagents and training programs. As a result, the impact and seasonality of influenza in tropical and subtropical areas of the world were being better defined so that it became possible, to say when and how long influenza transmits in particular areas.

Many countries began to appreciate that influenza was present and active over a good part of the year. Global Influenza Surveillance and Response System facilitated the dissemination of information between countries, including information on disease burden. This enabled countries without disease burden information to begin to appreciate the public health importance of influenza based on neighboring country or regional data. This also catalyzed countries to undertake their own studies, which in turn generated a greater demand for GISRS and GIP support.

## MOVING INTO THE POST‐PANDEMIC WORLD

The International Health Regulations had been put into effect shortly before the 2009 A(H1N1) pandemic establishing the critical role of WHO in the response.^15^ Global Influenza Surveillance and Response System and GIP played a central role during the pandemic, particularly focusing on issues such as severe disease occurring in pregnant women. The burden of seasonal influenza in pregnant women and their offspring resulted in recommendations for the use of influenza vaccines in pregnancy. In 2012, updates to the WHO vaccine risk group recommendations further strengthened the need to demonstrate burden. Without such demonstration, it will not be possible to convince much of the world that there is a need for seasonal vaccines, and without such use of seasonal vaccines, there will not be enough production capacity to supply the world with vaccines when the next pandemic occurs.

To facilitate countries to estimate their influenza disease burden and to build global estimates from such data, WHO issued a manual for estimating disease burden associated with seasonal influenza.^16^ Estimation is premised on surveillance systems that can distinguish laboratory‐confirmed disease from clinical syndromes. Many countries, and as evidenced in the studies represented in this issue, have relied on the work of their NICs to provide such information.

The next will be to demonstrate that vaccines can reduce severe disease. There is still, unfortunately, a belief in much of the world that influenza is a relatively mild, self‐limited illness. Countries with other major health issues will not take influenza prevention seriously unless it is demonstrated that there is significant, preventable severe morbidity and mortality, particularly in children under 2 years of age. This can be demonstrated in many ways, including a vaccine probe study that allows both the demonstration of the burden of severe disease and the ability of the vaccine used to prevent it.^17^ Results can then be further extrapolated to any new vaccines as development proceeds. With the increase in surveillance and laboratory testing under the GISRS umbrella in places where such severe illnesses are still common, it is now possible to conduct such studies, which, by demonstrating that burden is preventable, will have a long‐term effect on global control of influenza and its consequences. Now, it is our opportunity to reflect on the collective success, collaboration, and international efforts of GISRS and to congratulate the various institutions involved for their contribution to the study and control of a disease of global health importance. Happy birthday GISRS!
